# Improvement of Bacterial Blight Resistance in Two Conventionally Cultivated Rice Varieties by Editing the Noncoding Region

**DOI:** 10.3390/cells11162535

**Published:** 2022-08-16

**Authors:** Changyan Li, Lei Zhou, Bian Wu, Sanhe Li, Wenjun Zha, Wei Li, Zaihui Zhou, Linfeng Yang, Lei Shi, Yongjun Lin, Aiqing You

**Affiliations:** 1 Hubei Key Laboratory of Food Crop Germplasm and Genetic Improvement, Food Crops Institute, Hubei Academy of Agricultural Sciences, Wuhan 430064, China; 2 College of Life Science and Technology, Huazhong Agricultural University, Wuhan 430070, China; 3 Hubei Hongshan Laboratory, Wuhan 430070, China

**Keywords:** CRISPR/Cas9, molecular breeding, conventional rice, transgene-free, bacterial-blight-inducible element, noncoding region

## Abstract

*xa13* is a recessive pleiotropic gene that positively regulates rice disease resistance and negatively regulates rice fertility; thus, seriously restricting its rice breeding application. In this study, CRISPR/Cas9 gene-editing technology was used to delete the *Xa13* gene promoter partial sequence, including the pathogenic bacteria-inducible expression element. Rice with the edited promoter region lost the ability for pathogen-induced gene expression without affecting background gene expression in leaves and anthers, resulting in disease resistance and normal yield. The study also screened a family of disease-resistant and normal fertile plants in which the target sequence was deleted and the exogenous transgene fragment isolated in the T_1_ generation (transgene-free line). Important agronomic traits of the T_2_ generation rice were examined. T_2_ generation rice with/without exogenous DNA showed no statistical differences compared to the wild type in heading stage, plant height, panicles per plant, panicle length, or seed setting rate in the field. Two important conventional rice varieties, namely Kongyu131 (KY131, ***Geng***/*japonica*) and Huanghuazhan (HHZ, ***Xian***/*indica*), were successfully transformed, and disease-resistant and fertile materials were obtained. Currently, these are the two important conventional rice varieties in China that can be used directly for production after improvement. Expression of the *Xa13* gene in the leaves of transgenic rice (KY-PD and HHZ-PD) was not induced after pathogen infection, indicating that this method can be used universally and effectively to promote the practical application of *xa13*, a recessive disease-resistant pleiotropic gene, for rice bacterial blight resistance. Our study on the regulation of gene expression by editing noncoding regions of the genes provides a new idea for the development of molecular design breeding in the future.

## 1. Introduction

Gene-editing technology can delete, insert, or mutate target sites in a genome for the precise modification of genes. Since the 1990s, three generations of influential gene-editing techniques, including zinc-finger nucleases (ZFNs) [1], transcription activator-like effector nucleases (TALENs) [2], and clustered regularly interspaced short palindromic repeats/CRISPR-associated protein 9 (CRISPR/Cas9) [3], were developed. All these methods initiate non-homologous end joining (NHEJ) repair by inducing double-strand breaks at the DNA target site. Error-prone NHEJ repairs often result in base insertions or deletions at the repair site, resulting in target gene mutations. ZFNs and TALENs require a sequence-specific DNA binding module designed for each target DNA sequence to determine specificity in the binding site of the genome, while DNA cleavage activity is achieved by *Fok* I endonuclease fusion of ZFN and TALEN complexes. On the contrary, CRISPR/Cas9 technology uses a synthetic sgRNA-oriented molecule to identify a protospacer adjacent motif (PAM) with a (NGG)-specific DNA sequence, which is cleaved by the Cas9 protein. According to the research reports in various plant and animal models, CRISPR/Cas9 gene-editing technology is easier to operate, and more scalable, efficient, and stable than ZFNs and TALENs [4].

Rice is one of the most important food crops in the world. Since the successful use of gene-editing in plants, a large number of genes have been edited in rice, including traits such as yield, quality, resistance, and plant-architecture [5,6,7,8].

Rice bacterial blight caused by Gram-negative strains of *Xanthomonas oryzae* pv. *oryzae* (*Xoo*) is the most harmful bacterial disease affecting rice production [9]. The *xa13* gene is a cloned recessive bacterial blight resistance gene that also plays a key role in rice pollen development [10]. Analysis of the disease-resistance mechanism of the *xa13* gene showed that after bacterial infection, the dominant *Xa13* gene was activated and expressed which regulated the redistribution of sugar or copper ions leading to disease susceptibility [11,12]. RNAi-mediated constitutive silencing of the *Xa13* gene increased the ability of rice to resist bacterial blight but caused pollen sterility and a decreased seed setting rate. When the recessive *xa13* gene was mutated in the promoter region, the bacterial-blight-induced function was lost [10]. The 31-bp bacterial-blight-inducible element was verified in yeast and rice [13,14], and three different research groups have also obtained disease-resistant rice materials by editing this element [15,16,17].

Due to the evolution of pathogenic bacteria, a disease resistance gene often loses its resistance within 10 years of production. Compared to dominant genes, recessive resistance genes such as *xa13* may confer new resistance mechanisms against physiological pathogens that target crops and act as important supplements to dominant resistance gene resources [18]. However, in traditional crossbreeding, the use of recessive genes requires simultaneous modification of multiple parents, which is both time-consuming and laborious. Additionally, RNAi-mediated normal constitutive gene-silencing or knockout of the *Xa13* gene seriously affects rice pollen development and the seed setting rate. Though considered the most useful resistance gene in breeding, the unique mechanism of *xa13* disease resistance greatly limits its application in actual crop production.

In this study, two sgRNAs (P1 and P2) were designed using CRISPR/Cas9 gene-editing technology, and 149 nucleotides (including the 31 bp bacterial-blight-inducible element) were specifically deleted in the *Xa13* gene promoter region of two important popularized conventional rice varieties, Kongyu131 (KY131, ***Geng***/*japonica*) and Huanghuazhan (HHZ, ***Xian***/*indica*). Deletion of the 149 bp promoter fragment aimed to eliminate the induction of *Xa13* gene expression by bacterial blight without affecting anther development and rice fertility. The study also screened a family of disease-resistant and normal fertile plants in which the target sequence was deleted and the exogenous transgene fragment isolated in the T_1_ generation (transgene-free line). T_2_ generation transgenic plants with/without exogenous DNA showed no statistical differences in the main agronomic traits compared to the wild type. These findings proved that a successful deletion of the bacterial-blight-inducible element in the *Xa13* promoter did not affect rice agronomic traits except for disease resistance.

## 2. Materials and Methods

### 2.1. Selection of sgRNA Sites in the xa13 Gene Promoter

**Figure 1:**
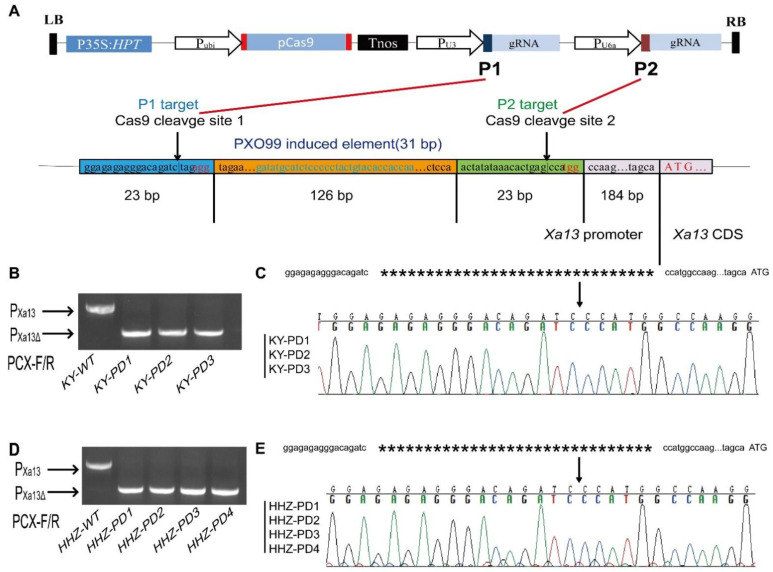
Schematic diagram of promoter editing vector Cas9: P1+P2 and results of the editing of the *Xa13* promoter mediated by Cas9: P1+P2 in two conventional rice varieties. (**A**) Schematic description of the wild-type *Xa13* promoter. Two target sites (P1 and P2) were located in the promoter region upstream of the *Xa13* CDS region. The 31 bp PXO99-inducible element was located entirely between the two target sites (P1 and P2). (**B**) PCR products amplified by primers PCX-F/R to detect the results of the *Xa13* promoter edited with the CRISPR/Cas9 system in the T_0_ generation of KY131. Three transgenic rice materials (KY-PD1-3), which were completely cut between P1 and P2 by Cas9 protein, were obtained. (**C**) Sequences and chromatograms of the three transgenic plants (KY-PD1-3) after editing. All three transgenic plants (PD1-3) were perfectly connected and repaired after cutting at the two target sites without any base deletion or insertion. (**D**) PCR products amplified by primers PCX-F/R to detect the results of the *Xa13* promoter edited with the CRISPR/Cas9 system in the T_0_ generation of HHZ. Four transgenic rice materials (HHZ-PD1-4), which were completely cut between P1 and P2 by the Cas9 protein, were obtained. (**E**) Sequences and chromatograms of the four transgenic plants (HHZ-PD1-4) after editing. All four transgenic plants (HHZ-PD1-4) were perfectly connected and repaired after cutting at the two target sites without any base deletion or insertion.

The *Xa13/xa13* gene promoter and CDS sequences were obtained from Chu et al. [10]. The 31 bp promoter bacterial-blight-induced element was obtained from Romer [13,14]. Two Cas9 target sites were selected upstream and downstream of the bacterial-blight-induced element. Target sequences were P1: actatataaacactgagcca***tgg*** and P2: ggagagagggacagatctag***agg*** (Figure 1A).

### 2.2. Cas9:P1+P2 Expression Vector Construction

Cas9:P1+P2 expression vector construction was performed according to the methods of Ma and Liu [19]. Based on P1 and P2 targets, four primers (P1U3-F, P1U3-R, P2U6a-F, and P2U6a-R) were designed for the Cas9:P1+P2 expression vector (Figure 1A). The primers used for Cas9:P1+P2 vector construction are listed in Table 1.

### 2.3. Transformation of Rice Expression Vector

The expression vectors were introduced into two rice varieties (KY131 and HHZ) using *Agrobacterium tumefaciens* strain EHA105. The callus culture and genetic transformation procedures of ***Geng***/*japonica* rice (KY131) and ***Xian***/*indica* rice (HHZ) were based on the methods described by Hiei [20] and Lin [21], respectively.

### 2.4. Transgenic Materials

The Cas9:P1+P2 vector was introduced into the ***Geng***/japonica rice (KY131) and ***Xian***/indica rice (HHZ). Three strains of mutants (KY-PD1-3) completely spliced between the P1 and P2 target sites in 41 T_0_ hygromycin-positive transformed KY131 plants were found (Figure 1B). Four strains of mutants (HHZ-PD1-4) completely spliced between the P1 and P2 target sites in 50 strain T_0_ hygromycin-positive transformed HHZ plants were found (Figure 1D). The primers used for molecular detection of transgenic rice plants in this study are listed in Table 2.

### 2.5. PCR Detection of the Xa13 Promoter Editing Sites

The genomic DNA of rice leaves of T_0_ generation was extracted using the CTAB method. The specific primers PCX-F/R were used to amplify the DNA of individual plants by PCR, and the transgenic rice with *Xa13* promoter editing were screened.

The PCR system was as follows: 10 × TaKaRa Taq (rTaq) buffer 2 μL, rice plant DNA 2 μL, forward primer 0.2 μM, reverse primer 0.2 μM, deoxyriboside triphosphate (dNTP) 10 s 0 μM, rTaq 0.2 μL, ddH_2_O added to 20 μL. PCR methods are as follows: 95 °C 2 min; 95 °C 30 s, 56 °C 30 s, 72 °C 30 s, 30 cycles; 72 °C 7 min. PCR products were detected by 0.8% agarose gel electrophoresis and mutated by Sanger method.

### 2.6. RT-PCR and qRT-PCR

Total RNA was isolated from anthers and leaves using TRIzol Reagent (Invitrogen, Carlsbad, CA, USA). Genome DNA was removed by DNase I (Promega, Madison, WI, USA).

Expression of *Xa13* and *gus* was determined by RT-PCR and qRT-PCR. RT was performed using a transcriptor First Strand cDNA Synthesis Kit (Roche, Mannheim, Germany), the RT-PCR was conducted using rTaqTM (TaKaRa, Dalian, China) with a 9700 PCR System (Applied Biosystems, Foster City, CA, USA), the RT-PCR products were separated on 1% agarose gel, and the qRT-PCR was conducted using SYBR^®^ Premix Ex Taq^TM^ (TaKaRa, Dalian, China) with a 7500 Real-Time PCR System (Applied Biosystems, Foster City, CA, USA).

### 2.7. Evaluation of Disease Resistance of Transgenic Rice Plants

We inoculated all the T_0_ transgenic plants in the field with the physiological race PXO99 of bacterial blight (using turbidimetric method to control the inoculum concentration between 900 million and 1.2 billion/mL) at the peak tillering stage. Each transgenic plant was inoculated with 5–6 leaves, inoculated by leaf-cutting method, and the length and area of lesions were investigated at 14 days after inoculation [22]. Data analysis was conducted with Duncan’s multiple testing in SPSS.

### 2.8. Evaluation of Fertility

When the T_0_ transgenic rice KY-PD1 and HHZ-PD1 were heading, the spikelets that were about to bloom were taken and placed in a centrifuge tube filled with 75% ethanol, and the samples were required to contain the upper, middle, and lower parts of the rice ear. Then, the anthers of spikelets were taken and placed on a glass slide carefully, 1 drop of I_2_-KI solution containing 0.1% (*w*/*v*) iodine and 1% (*w*/*v*) potassium iodide was added, then the anthers were crushed with tweezers, covered with a cover glass, and allowed to stand for 2–3 min for color development. Observations and recordings were taken using the microscope [23].

### 2.9. Examination of Resistance Inheritance and Cas9-Segregation in T_1_ Progeny

Cas9 PCR analysis was performed to determine the transgenic-free features of transgenic plants in T_1_ progeny (KY-PD1 and HHZ-PD1). Two T_1_ lines were grown in the greenhouse. Then, the resistance test at T_1_ seedling stage was carried out in the greenhouse. PXO99 was inoculated by cutting leaves at the 5-leaf stage of the seedling stage, and the phenotype was investigated at 14 days after inoculation [23].

### 2.10. Growth Curve of PXO99

The leaves of T_2_ progeny of KY-PD1 and HHZ-PD1 with/without Cas9 protein inoculated with bacterial blight were taken on 0, 2, 4, 6, 8, 10, 12, and 14 d, sterilized with 75% ethanol for 30 min, then the leaves were ground with 2 mL sterilized distilled water, The bacterial solution was diluted in sequence and divided into 7 gradients of 10^0^, 10^1^, 10^2^, 10^3^, 10^4^, 10^5^, and 10^6^, which were spread on potato medium plates for counting. The bacterial growth curve was made after counting the number of colonies [24].

## 3. Results

### 3.1. Cas9: P1+P2 Expression Vector Construction and Genotyping of Transgenic Plants

Based on the P1 and P2 targets of Cas9 in the *Xa13* promoter, the Cas9: P1+P2 expression vector was constructed (Figure 1A). Promoter editing vector Cas9: P1+P2 was transformed for two important conventional rice varieties in China, KY131 (***Geng***) and HHZ (***Xian***). Comparison of the sequencing results of the edited *Xa13* gene promoter regions of KY131 and HHZ showed the presence of one single nucleotide polymorphism (SNP) and two base insertions. These sequence differences were not present in the bacterial-blight-inducible element or the two gRNA target fragments, and thus the constructed vector was used for transformation.

Three T_0_ lines of KY-PD1-3 with complete shear between targets P1 and P2 (Figure 1B) were obtained. Sequencing results showed that the editing sites of KY-PD1, KY-PD2, and KY-PD3 were consistent with all the samples repaired by the third and fourth bases upstream of the P1 and P2 PAM sites (Figure 1C).

### 3.2. Detection of Xa13 Gene Expression in Transgenic Rice

RT-PCR/qRT-PCR detection primers (X13-Qt1F/R) were employed to detect the expression of the *Xa13* gene in the T_0_ generation of KY-PD1-3 and HHZ-PD1-4.

**Figure 2:**
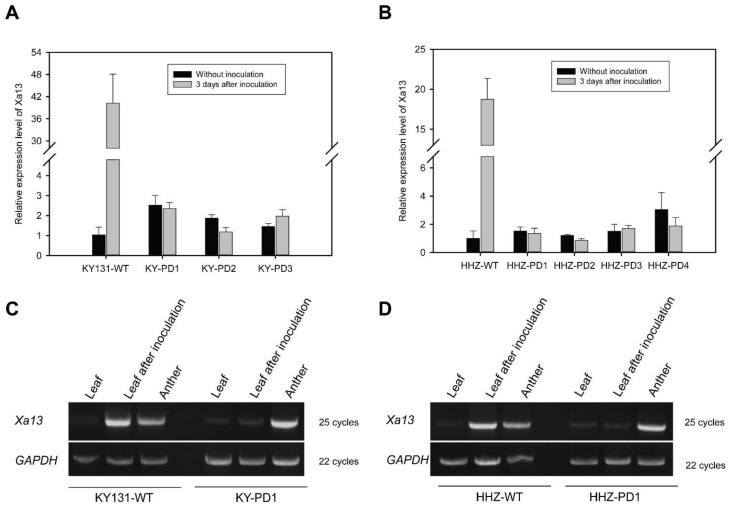
Detection of *Xa13* gene expression in T_0_ transgenic rice KY-PD1-3 and HHZ-PD1-4. (**A**) qRT-PCR analysis of *Xa13* in the leaves of transgenic rice KY-PD1-3 after PXO99 inoculation. Further detection of *Xa13* expression by qRT-PCR after inoculation in all T_0_ generation lines of KY-PD1-3. After inoculation, expression of *Xa13* was upregulated 40 times in the wild-type KY131, but not in transgenic rice KY-PD1-3. (**B**) qRT-PCR analysis of *Xa13* in the leaves of transgenic rice HHZ-PD1-4 after PXO99 inoculation. After inoculation, expression of *Xa13* was upregulated 19 times in the wild-type HHZ, but not in transgenic rice HHZ-PD1-4. (**C**) RT-PCR analysis of *Xa13* in the leaves and anthers of transgenic rice KY-PD1 by primer X13-Qt1F/R. In the wild-type KY131 and transgenic rice KY-PD1, expression levels of the *Xa13* gene were low in leaves but high in anthers. Expression of *Xa13* was greatly induced after PXO99 inoculation (3 days) in the wild-type KY131 but was quite low in transgenic rice KY-PD1. (**D**) RT-PCR analysis of *Xa13* in the leaves and anthers of transgenic rice HHZ-PD1 by primer X13-Qt1F/R. In the wild-type HHZ and transgenic rice HHZ-PD1, expression levels of the *Xa13* gene were very low in leaves but quite high in anthers. Expression of *Xa13* was greatly induced after PXO99 inoculation (3 days) in the wild-type HHZ but was quite low in transgenic rice HHZ-PD1.

The expression pattern of the *Xa1*3 gene before and after inoculation of T_0_ transgenic plants with KY-PD and HHZ-PD leaves was examined using qRT-PCR. The results showed that after inoculation, the expression of the *Xa13* gene in the wild-type KY131 and HHZ leaves was upregulated 40 and 18 times, respectively, while its expression had no significant change in the KY-PD1-3 and HHZ-PD1-4 transgenic plants (Figure 2A, B).

RT-PCR results showed that the expression of the *Xa13* gene in the wild-type KY131 and HHZ was lower in leaves but significantly increased after inoculation with PXO99. In addition, the *Xa13* gene was also highly expressed in anthers. Expression of the *Xa13* gene in leaves was low in the KY-PD1 and HHZ-PD1 samples and remained so after inoculation. Deletion of the 149 bp *Xa13* promoter fragment in the KY-PD and HHZ-PD samples did not affect the high expression levels of *Xa13* in anthers (Figure 2C, E).

These results demonstrated that KY-PD and HHZ-PD lacked the bacterial-blight-inducible element in the *Xa13* gene promoter as the *Xa13* gene in leaves was not induced by PXO99 infection and continued to maintain a low background expression level in leaves. However, the deletion of this element did not affect *Xa13* gene expression in anthers.

### 3.3. Disease Resistance and Agronomic Traits of T_0_ Generation KY-PD1-3 and HHZ-PD1-4

**Figure 3:**
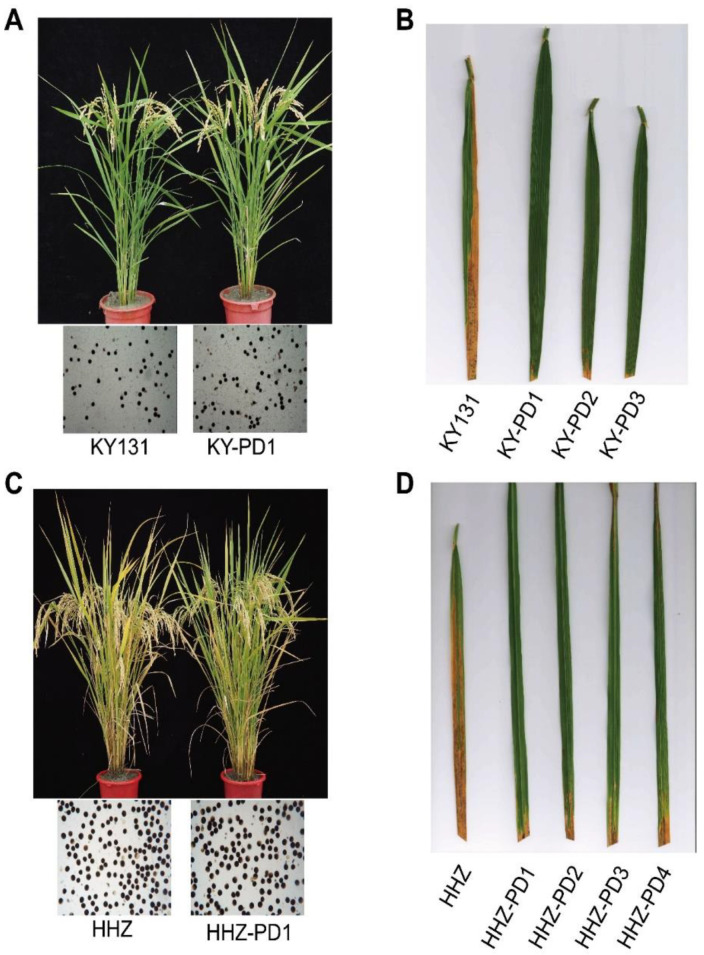
Agronomic traits, bacterial leaf blight resistance, and pollen fertility of wild-type and transgenic rice plants (KY-PD1 and HHZ-PD1). (**A**) Agronomic traits and phenotype detection of the wild-type KY131 and transgenic rice KY-PD1. There was no significant difference between transgenic rice KY-PD1 and the wild type in terms of agronomic traits. Transgenic plants KY-PD1 showed similar pollen fertility to the wild-type KY131 after I_2_-KI staining of pollen. (**B**) Lesion length of the wild-type KY131 and transgenic plant KY-PD1-3 after 14 days of inoculation. Resistance of transgenic plants KY-PD1-3 was much higher than that of the wild-type KY131. (**C**) Agronomic traits and genotype detection of the wild-type HHZ and transgenic rice HHZ-PD1. There was no significant difference between transgenic rice HHZ-PD1 and the wild type in terms of agronomic traits. Transgenic plants HHZ-PD1 showed similar pollen fertility to the wild-type HHZ after I_2_-KI staining of pollen. (**D**) Lesion length of the wild-type HHZ and transgenic plant KY-PD1-3 after 14 days of inoculation. Resistance of transgenic plants HHZ-PD1-4 was much higher than that of the wild-type HHZ.

Wild-type KY131 was examined at 14 days after PXO99 inoculation. Average leaf lesion length and lesion area of the wild-type KY131 were 16.99 ± 4.63 cm and 62.46 ± 12.90% (*n* = 50, Table 3), respectively. In contrast, the T_0_ generation samples of KY-PD1-3 showed strong PXO99 resistance (Figure 3B) with lesion length and lesion area differing significantly from the wild-type KY131 (Table 3).

Although *Xa13* mutants and RNAi-mediated constitutively silenced *Xa13* transgenic plants showed enhanced disease resistance, pollen development was severely inhibited in them, resulting in sterile phenotypes [10,17]. Analysis of KY-PD1 and HHZ-PD1 agronomic traits in the T_0_ plants of our study showed that they were not significantly different from the phenotype of the wild-type families (Figure 3A,C).

Staining tests showed that the anthers of KY-PD and HHZ-PD were stained with potassium iodide; thus, indicating normal anther development (Figure 3A,C). Additionally, KY-PD1-3 and HHZ-PD1-4 showed no significant difference in the seed setting rates when compared between PD and the wild type (Table 3).

### 3.4. Screening of Cas9-Free Plants from the T_1_ Generation Single-Copy PD1 Family

Analyses of the agronomic traits of KY-PD1 and HHZ-PD1 were not significantly different from the wild type (Figure 3A,C). Therefore, KY-PD1 and HHZ-PD1 were chosen for the following T_1_ generation analysis. At the seedling stage of the T_1_ generation plants of KY-PD1 and HHZ-PD1, PCR was performed on the *Cas9* gene to report a negative family (transgenic-free).

**Figure 4:**
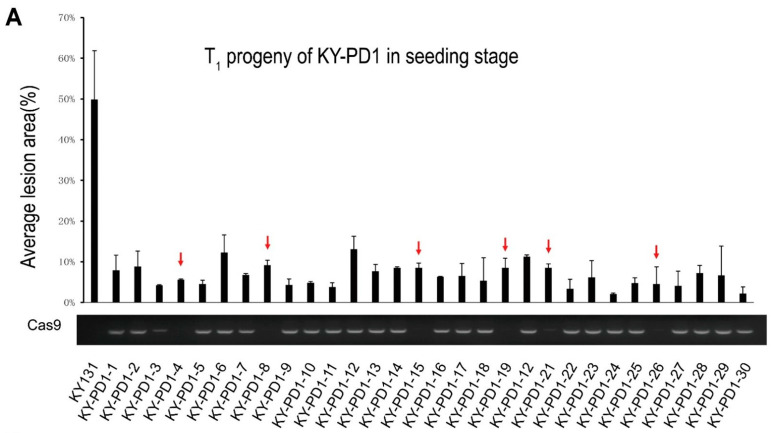

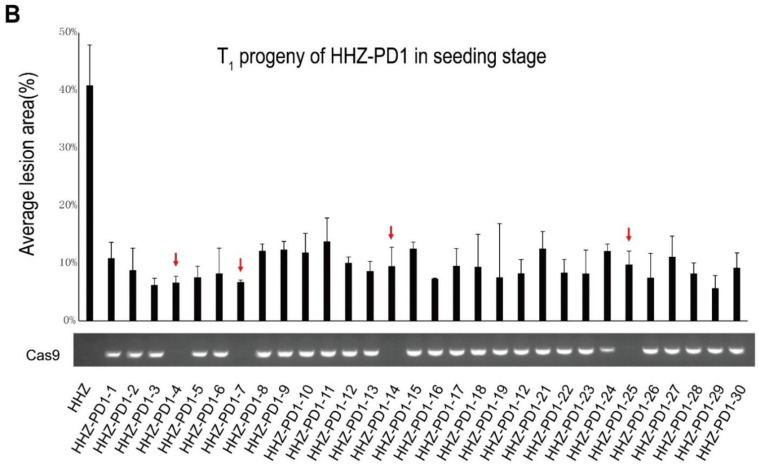
Disease resistance and genotype identification test in T_1_ progeny of KY-PD1 and HHZ-PD1. (**A**) Average lesion area of each T_1_ plant was measured 14 days after PXO99 inoculation to evaluate disease resistance. Compared with KY131-WT, all T_1_ progeny of KY-PD1 showed good disease resistance to PXO99. Results of PCR by primers PCas9-F/R showed that 6 of the 30 T_1_ plants failed to detect the Cas9 sequence. (**B**) Average lesion area of each T_1_ plant was measured 14 days after PXO99 inoculation to evaluate disease resistance. Compared with HHZ-WT, all T_1_ progeny of HHZ-PD1 showed good disease resistance to PXO99. Results of PCR by primers PCas9-F/R showed that 4 of the 30 T_1_ plants failed to detect the Cas9 sequence. The red arrow indicates transgene-free T_1_ plants without exogenous Cas9-introduced fragment.

Resistance of the T_1_ generation plants of the KY-PD1 family was identified at the seedling stage. The results showed that the percentage of control lesions in KY131 was approximately 49.85%, while it was below 15% in both *Cas9*-positive and transgene-free *Cas9*-negative T_1_ generation plants of the KY-PD1 family (red arrow). The results indicated that the leaf blight disease resistance of the KY-PD1 family was stable and inherited (Figure 4A). Six Cas9-negative KY-PD1 families (red arrows, transgene-free) were successfully isolated in 30 T_1_ generation KY-PD1 samples.

### 3.5. Investigation of Important Agronomic Traits in the T_2_ Generation of Transgene-Free PD1 Family and Identification of the Bacterial Growth Curve

**Figure 5:**
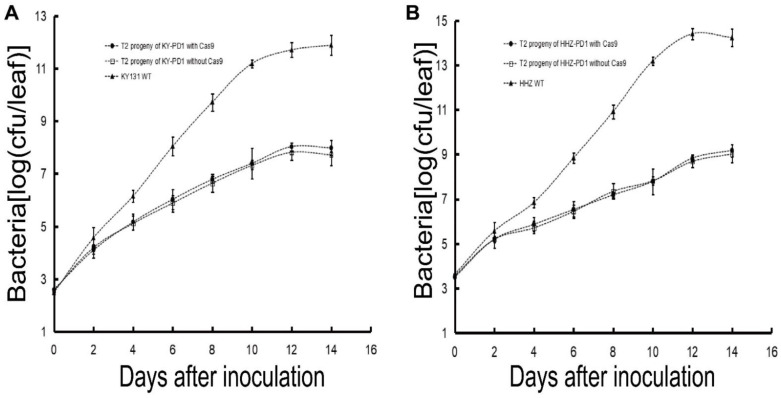
Bacterial growth analysis of *Xoo* strain PXO99 in leaves of wild-type and transgenic rice with/without Cas9 in T_2_ progeny. (**A**) Bacterial growth analysis of *Xoo* strain PXO99 in the leaves of wild-type KY131, T_2_ transgenic line KY-PD1 with Cas9, and T_2_ transgenic line KY-PD1 without Cas9. The results revealed that both PXO99-inoculated KY-PD1 with/without exogenous DNA showed lower growth rates and total bacterial counts than the wild-type KY131. (**B**) Bacterial growth analysis of *Xoo* strain PXO99 in the leaves of wild-type HHZ, T_2_ transgenic line HHZ-PD1 with Cas9, and T_2_ transgenic line HHZ-PD1 without Cas9. The results revealed that both PXO99-inoculated HHZ-PD1 with/without exogenous DNA showed lower growth rates and total bacterial counts than the wild-type HHZ.

KY-PD1 and HHZ-PD1 samples with/without exogenous DNA were used to map the bacterial growth curve after PXO99 infection. The results showed that the T_2_ generation plants (with/without exogenous DNA) of both PXO99 inoculated KY-PD1 and HHZ-PD1 showed lower growth rates and total bacterial counts compared to the wild type. This indicated that bacterial growth was inhibited in the KY-PD1 and HHZ-PD1 families after PXO99 inoculation due to the deletion of the bacterial-inducible element (Figure 5).

Important agronomic traits of the T_2_ generation plants belonging to the KY-PD1 and HHZ-PD1 families were examined (Table 4 and Table 5). T_2_ generation KY-PD1 and HHZ-PD1 plants with/without exogenous DNA showed no statistical differences compared to the wild type in heading stage, plant height, panicles per plant, panicle length, or seed setting rate. This proved that the successful deletion of the bacterial-blight-inducible element in the *Xa13* gene promoter did not affect rice agronomic traits except for disease resistance.

Promoter editing vector was successfully used in the present study to improve bacterial blight resistance in two conventionally cultivated rice varieties, KY131 (***Geng***) and HHZ (***Xian***). This method of editing the promoter region can be used universally and has great application potential in breeding disease-resistant rice, especially for conventional rice varieties.

Duncan’s multiple testing between KY-PD1 with/without exogenous DNA and KY131, a and b were ranks resolved by least significant difference (LSD) method at the 0.05 probability levels. A and B were ranks resolved by least significant difference (LSD) method at the 0.01 probability levels. Data in the same column with the same letter meant there was no significant difference between them.

Duncan’s multiple testing between HHZ-PD1 with/without exogenous DNA and HHZ, a and b were ranks resolved by least significant difference (LSD) method at the 0.05 probability levels. A and B were ranks resolved by least significant difference (LSD) method at the 0.01 probability levels. Data in the same column with the same letter meant there was no significant difference between them.

## 4. Discussion

### 4.1. Advantages and Limitations of Changing Target Gene Expression Pattern by Editing the Promoter Region

At present, most gene-editing target sites lie in the coding sequence (CDS) region. However, they lead to frameshift mutations or erroneous mRNA or protein. By editing a noncoding region, additional mRNA and protein production can be avoided. Additionally, certain pluripotent genes such as *Xa13* cannot be used in the editing of the CDS region.

Rodriguez-Leal et al. used CRISPR/Cas9 to cause multiple site mutations of three yield-related gene promoters in tomato plants. This produced multiple sets of mutations leading to a series of continuous and unexpected mutant tomato plants. Series of continuous mutations provide a rich source of variations for breeding [25].

TALENs were used by two research groups to edit the promoter of the *OsSWEET14* gene in rice to obtain disease-resistant rice phenotypes [26,27]. Elsewhere, CRISPR/Cas9 was used to edit the *CsLOB* gene promoter in citrus to produce disease-resistant citrus fruits [28,29]. In the above studies, single-target mutations were used for random repair, and as a result, a variety of different mutations or even different allele mutations in the same transgenic plant were obtained which shall be discussed in Section 4.2.

Our study proposed to delete the bacterial-blight-inducible element in the promoter region of the dominant disease-susceptibility gene *Xa13* in rice to evade the ability of the *Xa13* gene to be induced by the pathogen and display a disease-resistant phenotype. The results showed that the expression of *Xa13* gene in the leaves of transgenic rice (KY-PD and HHZ-PD) was not induced after pathogen infection. Moreover, element deletion did not reduce *Xa13* gene expression in anthers, and hence did not affect fertility and seed setting rates.

Theoretically, when a promoter element function is clear and the PAM site is appropriate, the gene expression pattern can be accurately changed by editing the promoter regulatory element of the gene. The biggest limiting factor in this technology is the selection of the PAM site. Hu et al. successfully verified the latest version of the xCas9 protein obtained by the directed evolution method in human cells. This protein has three PAM sites (NG, GAA, and GTA) compared to the currently used spCas9 protein, which has only one PAM locus (NGG). This greatly increases the selection range for editing targets [30]. If the xCas9 protein was found to have the same gene-editing function and efficiency in rice, the promoter deletion fragment in our study could have been strictly controlled to a shorter fragment size.

Recent research on the 5’ untranslated region (UTR) of the *GGP2* gene in lettuce edited by the CRISPR/Cas9 system obtained high translation efficiency in the *GGP2* gene, which in turn greatly increased vitamin C content in lettuce leaves [31].

Editing of the promoter regions simply changes the expression pattern of genes without producing frameshift mutations or erroneous mRNA or protein which ensures truly transgene-free material. Improvement in crop traits by editing noncoding regions will soon be of great importance in the future.

### 4.2. Advantages of CRISPR/cas9-Mediated Double-Target sgRNA Spot Deletion

Most *cas9* gene knockouts are used for single-target random mutations, resulting in a small number of base deletions, insertions, mutations, or single-base mutations. It is possible to even have allele mutations of different genotypes in the same transgene plant [28,29]. In most cases, these mutations cannot be detected by PCR and require identification by large-scale sequencing, which is time-consuming, labor-intensive, and costly. Double sgRNA-mediated deletion mutation is relatively stable compared to the randomness of a single target-induced mutation (insertion, deletion, or mutation), and the mutation results are predictable. All seven homozygous mutated transgenic rice lines (KY-PD1-3 and HHZ-PD1-4) obtained in this study showed similar results concerning the expected shear conditions. A double-sgRNA site-directed deletion mutation was used to select the target site in this study for the following reasons: (i) it was directed at DNA sequence deletion, (ii) it was easy to use PCR to identify whether the mutation site was a homozygous, heterozygous, or no deletion mutation, (iii) it greatly reduced the sequencing workload, and (iv) it improved the accuracy of selecting mutant plants.

### 4.3. Application of Recessive and Pleiotropic Gene xa13 in Rice Disease Resistance Breeding

The *xa13* gene is a typical multi-effect gene with major recessive bacterial blight resistance that controls disease resistance and regulates pollen development in rice.

As the use of recessive resistance genes is inconvenient in rice breeding, breeders are more concerned with the application of dominant resistance genes. Therefore, it is crucial to make full use of recessive resistance genes in the long-term struggle between human-mediated plant production and pathogenic bacteria.

Breeding practices demonstrate that the *xa13* gene is not only resistant to the *Xoo* strain PXO99, but most other *Xoo* strains as well. This calls for increased attention to the application of the recessive *xa13* gene in breeding [32]. However, in conventional breeding, especially crossbreeding, the use of recessive genes is relatively time-consuming and laborious, which restricts the use of the *xa13* gene in actual production.

To study the effective use of the recessive disease resistance gene *xa13*, a green tissue-specific expression promoter was used to drive an artificial microRNA (amiRNA) in our previous study which was introduced into the rice restorer ‘Minghui 63’ [23]. This successfully transformed the recessive disease resistance trait into a dominant trait. Thus, in hybrid crossbreeding practice, only one of the parents is required to be modified to prepare a disease-resistant hybrid seed. Additionally, the green tissue-specific promoter was used to limit the silencing of the *Xa13* gene in the green tissue without affecting its expression in the anther, thereby avoiding a decline in fertility. This study effectively improved the application of the *Xa13* gene in disease-resistant crossbreeding practice [23].

In addition to the above-mentioned regulation of *Xa13* at the transcription level to achieve disease resistance, *Xa13* can be edited at the genome level. In the present study, rice materials KY131 and HHZ with high resistance to bacterial blight and no effect on seed yield were obtained by editing the inducible expression elements of the susceptible *Xa13* gene promoter. This proved that the proposed method has great potential in practical disease-resistant breeding applications, especially for conventional rice varieties.

Furthermore, unlike traditional RNAi silencing technology, which relies on the *trans*-acting effects of exogenously introduced fragments to play different roles, the CRISPR/Cas9 genome-editing technique can isolate a transgene-free plant in the offspring after successful editing of the target fragment. With a genetic background similar to natural mutants, crops modified with CRISPR/Cas9 can be readily accepted by the public, especially those edited at the gene promoter region by a technique that does not produce erroneous or additional mRNAs and proteins.

## 5. Conclusions

In the present study, a bacterial blight-inducible element was specifically deleted in the *Xa13* gene promoter regions of two important conventional rice varieties, KY131 (***Geng***) and HHZ (***Xian***), by Cas9 with two sgRNAs. The deletion of the bacterial blight-inducible element aimed to prevent the induction of the *Xa13* gene expression by bacterial blight with no effects on anther development and rice fertility. The proposed method of editing the promoter region was successful in improving the bacterial blight disease resistance of KY131 and HHZ and can be used universally in disease-resistant breeding applications, especially for conventional rice varieties. Regulating gene expression by editing noncoding regions of the genes provides a new idea for the development of molecular design breeding in the future

## Figures and Tables

**Table 1:** List of the primers used for CRISPR-Cas9 expression vector construction.

Primer	Primer Sequence (5’-3’)	Function
P2U6a-F	GCCGgagagagggacagatctag	vector construction
P2U6a-R	AAACctagatctgtccctctctc	vector construction
P1U3-F	GGCActatataaacactgagcca	vector construction
P1U3-R	AAACtggctcagtgtttatatag	vector construction
gRctga-B2	AGCGTGggtctcGtcagGGTCCATCCACTCCAAGCTC	vector construction
Uctga-B2′	TTCAGAggtctcTctgaCACTGGAATCGGCAGCAAAGG	vector construction
gRcggt-BL	AGCGTGggtctcGaccgACGCGTCCATCCACTCCAAGCTC	vector construction
U-F	CTCCGTTTTACCTGTGGAATCG	vector construction
gRNA-R	CGGAGGAAAATTCCATCCAC	vector construction
PT1	5’-TTCAGAggtctcTctcgACTAGTGGAATCGGCAGCAAAGG-3’/5’-AGCGTGggtctcGtcagGGTCCATCCACTCCAAGCTC-3’	vector construction
PT2L	5’-TTCAGAggtctcTctgaCACTGGAATCGGCAGCAAAGG-3’/5’-AGCGTGggtctcGaccgACGCGTCCATCCACTCCAAGCTC-3’	vector construction

**Table 2:** List of the primers used for molecular detection of transgenic rice.

Primer	Primer Sequence (5’-3’)	Function
PCX-F	GCATCATTGTCCATGGTTG	genotype test
PCX-R	GCTAGAGAGGAAGGCTTAAG	genotype test
GAPDH-F	CTGCAACTCAGAAGACCGTTG	qRT-PCR test
GAPDH-R	CCTGTTGTCACCCTGGAAGTC	qRT-PCR test
X13-Qt1F	AGTCGACGGGAGGGTACAG	qRT-PCR test
X13-Qt1R	GACGAGGTAGAGGGTGGTGA	qRT-PCR test
PCas9-F	ATGACTCTCTTACCTTCA	genotype test
PCas9-R	TAGTTCTTCATCTTCTTG	genotype test
hpt-F	AGAATCTCGTGCTTTCAGCTTCGA	genotype test
hpt-R	TCAAGACCAATGCGGAGCATATAC	genotype test

**Table 3:** Disease resistance and agronomic traits of T_0_ generation KY-PD1-3 and HHZ-PD1-4 plants.

Line	n	Lesion Length (cm) ^a^	Lesion Area (%) ^a^	Seeding Setting Rate (%)
KY131	50 ^b1^	16.99 ± 4.63	62.46 ± 12.90	77.5 ± 10.3 ^c^
KY-PD1	6	1.25 ± 0.53	5.32 ± 1.93	74.6
KY-PD2	6	1.33 ± 0.68	6.46 ± 3.67	67.4
KY-PD3	6	0.83 ± 0.52	3.00 ± 2.08	81.1
HHZ	50 ^b2^	17.92 ± 4.73	65.94 ±13.60	89.6 ± 3.7 ^c^
HHZ-PD1	6	1.75 ± 0.42	7.56 ± 1.65	86.38
HHZ-PD2	6	1.58 ± 0.38	7.36 ± 2.36	88.28
HHZ-PD3	6	1.50 ± 0.32	5.30 ± 1.67	93.46
HHZ-PD4	6	2.00 ± 0.32	7.03 ± 1.46	89.83

^a^ Data are means ± standard deviation; ^b^ 50 leaf samples from 10 plants; ^c^ 10 plants.

**Table 4:** Main agronomic traits of KY-PD1 in the T_2_ generation.

Line	*n*	Heading Stage (d)	Plant Height (cm)	Panicles per Plant	Panicle Length (cm)	Seeding Setting Rate (%)	Yield per Plant (g)
KY131	30	64.40 ± 1.35aA	81.73 ± 1.60aA	9.73 ± 1.64aA	19.32 ± 0.62aA	79.25 ± 5.09aA	20.44 ± 1.00aA
KY-PD1 transgenic lines	30	65.13 ± 1.65aA	82.23 ± 1.41aA	9.57 ± 1.61aA	19.08 ± 0.48aA	78.66 ± 5.65aA	20.17 ± 0.91abA
KY-PD1 transgenic-free lines	30	65.13 ± 1.93aA	82.17 ± 1.60aA	10.13 ± 0.90aA	19.01 ± 0.62aA	79.13 ± 4.14aA	20.72 ± 0.97bA

**Table 5:** Main agronomic traits of HHZ-PD1 in T_2_ generation.

Line	*n*	Heading Stage (d)	Plant Height (cm)	Panicles per Plant	Panicle Length (cm)	Seeding Setting Rate (%)	Yield per Plant (g)
HHZ	30	93.60 ± 1.81aA	101.90 ± 2.35aA	16.67 ± 1.63aA	22.42 ± 0.70aA	84.71 ± 5.29aA	36.26 ± 1.20aA
HHZ-PD1 transgenic lines	30	93.10 ± 2.87aA	101.00 ± 2.79aA	16.57 ± 1.61aA	22.30 ± 0.46aA	84.29 ± 5.64aA	36.07 ± 0.81abA
HHZ-PD1 transgenic-free lines	30	92.27 ± 3.30aA	101.57 ± 1.92aA	16.93 ± 1.05aA	22.25 ± 0.49aA	85.01 ± 4.15aA	36.65 ± 0.87bA

## Data Availability

Not applicable.
